# Loss of E-Cadherin Staining Continuity in the Trophoblastic Basal Membrane Correlates with Increased Resistance in Uterine Arteries and Proteinuria in Patients with Pregnancy-Induced Hypertension

**DOI:** 10.3390/jcm11030668

**Published:** 2022-01-27

**Authors:** Marta Pęksa, Alexandra Kamieniecki, Anna Gabrych, Anna Lew-Tusk, Krzysztof Preis, Małgorzata Świątkowska-Freund

**Affiliations:** 1 Department of Obstetrics, Medical University of Gdańsk, 7 Debinki Street, 80-211 Gdansk, Poland; kpreis@gumed.edu.pl (K.P.); malgorzata.swiatkowska-freund@gumed.edu.pl (M.Ś.-F.); 2 Department of Pathomorphology, Medical University of Gdańsk, 7 Debinki Street, 80-211 Gdansk, Poland; ola_kamieniecki@gumed.edu.pl; 3 Department of Pediatrics, Hematology, and Oncology, Medical University of Gdansk, 7 Debinki Street, 80-211 Gdansk, Poland; anna.gabrych@gumed.edu.pl; 4 Department of Neonatology, St. Adalbert’s Hospital, 80-462 Gdansk, Poland; ano.lew@wp.pl

**Keywords:** placenta, E-cadherin, epithelial-mesenchymal transition, ultrasound, preeclampsia, hypertension, pregnancy

## Abstract

Pregnancy-induced hypertension (PIH), especially when complicated with pre-eclampsia (PE), could be a life-threatening complication of pregnancy. Pre-eclampsia is one of the leading causes of perinatal morbidity and mortality in women. Pre-eclampsia is mainly characterized by hypertension and kidney damage with proteinuria. Abnormal placentation and altered structure of the placental barrier are believed to participate in the pathogenesis of pregnancy-induced hypertension, leading to PE. In the current study, we aimed to analyze the immunohistochemical expression pattern of E-cadherin and p120, two markers of epithelial–mesenchymal transition, in placental samples derived from a group of 55 patients with pregnancy-induced hypertension, including pre-eclampsia and 37 healthy pregnant controls. The results were correlated with the presence of an obtained early uterine artery flow notching during diastole on Doppler ultrasound. We observed a higher frequency of discontinuous E-cadherin staining in the basement membrane of syncytiotrophoblast in patients with PIH/PE compared to controls (*p* < 0.001, Fisher’s exact test). Moreover, the loss of continuity of E-cadherin expression correlated with the presence of a bilateral early diastolic notch on Doppler ultrasound (*p* < 0.001, Fisher’s exact test) and the presence of proteinuria (*p* = 0.013, Fisher’s exact test). These findings suggest that E-cadherin contributes to the integrity of the placental barrier, and its loss could be an immunohistochemical marker of PE.

## 1. Introduction

Pre-eclampsia (PE) is a complication in 2–5% of pregnancies and is one of the leading causes of perinatal morbidity and mortality in women, especially when it has an early onset. It is more common in developing countries due to older maternal age and obesity [1,2]. There is still no agreement on the definition of pre-eclampsia amongst many guidelines [2]. PE is defined by the International Society for the Study of Hypertension in Pregnancy (ISSHP) by hypertension (blood pressure ≥ 140/90 mmHg) developing after 20 weeks’ gestation coexisting with newly occurring proteinuria and/or maternal kidney damage [2]. Modern definitions acknowledge other manifestations including maternal liver dysfunction, neurological symptoms, hemolysis, thrombocytopenia, and uteroplacental dysfunction, such as an abnormal umbilical artery Doppler [2,3,4,5,6]. Some guidelines recommend a combined screening strategy including maternal blood pressure, maternal factors, uterine artery doppler, and placenta growth factor levels [2]. Treatment recommendations vary on whether non-severe hypertension should even be treated, although there is an overall agreement on the drugs of choice (methyldopa, labetalol, and nifedipine), and the timing of delivery in uncomplicated gestational hypertension cases, which should be on or after 37 weeks of gestation [2].

The placenta is the most important organ in the development of pregnancy. As early as the first trimester, the establishment of sufficient maternal–fetal circulation through correct implantation is crucial for further fetal development. The pathogenesis of various pre-eclampsia presentations has not been fully understood [7]. The trophoblast, penetrating the uterine mucosa, differentiates into the inner layer, the cytotrophoblast, and the outer layer, the syncytiotrophoblast, which lines the entire surface of the developing placenta, together forming the chorionic villi [8]. The distal villous cytotrophoblast transforms into cell column cytotrophoblast, which ultimately acquires a mesenchymal phenotype and starts to invade maternal decidua as interstitial extravillous trophoblast (EVT) [9]. The transformation of the trophoblast cell column into migratory cells of EVT is possible due to the epithelial–mesenchymal transformation (EMT), a process in which epithelial cells acquire mesenchymal cell properties, including the ability to migrate and invade. This process anchors the placenta to maternal tissues and plays a role in vascular remodeling [10]. Epithelial cadherin (E-cadherin) is a key phenotypic marker of cells in the epithelial state [11]. It is a transmembrane glycoprotein responsible for the maintenance of intercellular connections. P120-catenin is a protein that binds to the cytoplasmic perimembranous domain of classic cadherins and regulates their stability [11]. Loss of E-cadherin expression and localization shifts of p120 from cell–cell junctions to the cytoplasm are hallmarks of EMT [12]. During pregnancy, E-cadherin expression in trophoblastic cells decreases temporarily during the placental invasion in the first and second trimesters of pregnancy [13]. As the placenta matures, the functional localization of E-cadherin is restored. Through vascular remodeling, the maternal vessels, which are characterized by high resistance before pregnancy, are remodeled into vessels with a wide lumen and low resistance [14]. The conversion of the spiral arteries into low resistance uteroplacental vessels enables adequate nourishment of the fetus early on in pregnancy. Disturbances in the control of migration and trophoblast invasion in pregnancy are believed to contribute to several pregnancy pathologies. Shallow invasion is a hallmark of pregnancy-induced hypertension (PIH) and PE [15].

Abnormal trophoblast implantation leading to a reduction in blood flow in the uteroplacental circulation can be visualized by examining the uterine arteries via Doppler ultrasound [16]. The increased resistance and reduced vascular compliance in the uteroplacental circulation translate to an increase in the pulsatility index (PI) and an abnormal Doppler waveform in the uterine arteries [17,18]. The presence of an early diastolic notch (defined as a reduction in flow by 50 Hz from the maximum diastolic velocity) at the border of the systolic and diastolic parts correlates with decreased vascular compliance resulting from incomplete trophoblast invasion into the spiral arteries in the first and second trimesters of pregnancy [19]. Importantly, uterine artery notching is a normal finding in the gravid uterus up to 13–16 weeks of pregnancy. On the other hand, the early diastolic notch in late pregnancy (after 22 weeks) reflects abnormal blood flow in the uteroplacental circulation and predicts adverse perinatal outcomes. Unilateral notching may be simply associated with placental laterality; thus, bilateral notching is a more valuable risk predictor. Due to its low interobserver variability, it is a reliable method in the assessment of uteroplacental circulation [19].

The pathogenesis of PIH/PE remains vague. We hypothesize that the altered expression of p120 and E-cadherin in the basal membrane of the syncytiotrophoblast in the late second and third trimesters of pregnancy may be markers of the disruption of the placental barrier. The current prospective case-controlled study aimed to assess the condition and continuity of the placental barrier by using immunohistochemical markers, E-cadherin, and p120 in patients with PIH/PE compared to a healthy pregnant control group in the context of ultrasound evaluation of the uteroplacental circulation and the measurement of proteinuria.

## 2. Materials and Methods

### 2.1. Study Group

The study included a group of 55 Caucasian females hospitalized due to pregnancies complicated by PIH/PE at the Obstetrics and Gynecology Department of St. Wojciech’s Hospital in Gdansk, Poland, between 2012 and 2013 and whose stay in the Department resulted in childbirth. The inclusion criteria of the study group was a singleton pregnancy in the absence of other comorbidities. The control group consisted of 37 pregnant Caucasian women who gave birth without complications at the St. Wojciech hospital in Gdansk. The group included healthy female patients with a singleton pregnancy.

Information about patients’ age and the level of proteinuria at the time of admission were retrieved from past medical records.

### 2.2. Ultrasound

An ultrasound examination was performed on each patient included in the study. The examination was performed shortly before labor. Uterine artery velocity waveforms were recorded via transabdominal sonography utilizing the SIEMENS ACUSON X500 and a probe with a frequency of 3.5 MHz. Color-coded Doppler flow mapping was used to identify each uterine artery after visualizing the crossover of the uterine artery with the external iliac artery in the right and left iliac fossa. A pulsed-wave Doppler was used with the sampling gate set at 5 mm, which was placed less than 4 cm from the crossover point with the angle of insonation of <30° to provide accurate and reliable measurements. The gate size corresponded to the size of the examined arteries. For the uterine arteries spectral waveform analysis, automatic or manual tracing of the waveforms was performed to generate the Doppler parameters. The presence of an early diastolic notch was used as an ultrasound indicator of abnormal uterine artery flow.

### 2.3. Immunohistochemistry

Microscopic slides from each placenta were reviewed by a pathologist for the selection of representative areas of interest. Subsequently, tissue microarrays (TMAs) were created with the Beecher Instruments Manual Tissue Arrayer I (MTAI, K7 BioSystems) using 1.5 mm core needles. For each case, 3 cores (from the maternal, central, and fetal parts of the placenta) were included. Subsequently, immunohistochemical staining was performed on tissue sections of 4 µm previously prepared TMAs mounted on silanized slides (Surgipath), and then, routine dewaxing was done. Obtained slides were stained with antibodies against E-cadherin (clone NCH-38, catalog number IR 059) and p120 (clone Catenin Ventana). All antibodies used were ready to use. E-cadherin staining was performed on a DAKO Autostainer Link device and p120 staining was performed on a Benchmark GX device.

The E-cadherin expression in the basement membrane of the syncytiotrophoblast was assessed based on a three-point scale based on the intensity of the reaction: 1—weak reaction; 2—medium reaction; 3—strong reaction. The assessment of the continuity pattern of E-cadherin expression in the basement membrane of the syncytiotrophoblast was performed based on a two-point scale: 0—discontinuous staining; 1—continuous staining. Assessment of the intensity of the p120 protein in the membrane and cytoplasm of the chorionic cells, and the endothelium of the placental vessels was made based on a three-point scale: 0—no reaction; 1—weak reaction; 2—medium reaction.

### 2.4. Statistics

The obtained results of the immunohistochemical assessment of the placenta were correlated with the results of the ultrasound and laboratory tests. Statistical analysis was performed using the R statistical environment (version 4.0.0) [20]. The association of frequencies in qualitative variables was analyzed using the Chi-square test or the Fisher test, depending on the size of the subgroups. *p* values less than α = 0.05 were considered statistically significant. The ggplot2 package was used for the visualization of data [21]. The comparison of qualitative variables between the groups was visualized by using stacked bar charts.

### 2.5. Ethics

The study was approved by the Bioethical Committee of Medical University of Gdańsk (approval No NKEBN/75/2011).

## 3. Results

### 3.1. Basic Characteristics of the Study and Control Groups

The mean age of patients in the study group was 30.1 years (standard deviation, SD = 5.53), and in the control group, it was 28.1 (SD = 4.52). The difference between groups in terms of maternal age was statistically insignificant (*p* = 0.117, Mann–Whitney *U*-test). The median gestational week of delivery was 37 (range 28–42) in the study group compared to 40 (range 38–42) in the control group and significantly differed between groups (*p* < 0.001, Mann–Whitney *U*-test). Median birth weight was 2160 g in the study group and 3475 g in the control group (*p* < 0.001, Mann–Whitney *U*-test). Basic categorical characteristics are presented in Table 1.

### 3.2. Incidence of an Early Diastolic Notch and Proteinuria in the Study and Control Groups

The early diastolic notch was significantly more frequent in the study group than in the control group. The results were similar in terms of both unilateral (37% vs. 2.7%, *p* < 0.001, Fisher’s exact test) and bilateral notching (40.7% vs. 2.7%, *p* < 0.001, Fisher’s exact test). Similar associations were observed if the study group was restricted to gestational age ≥32 weeks (bilateral notching—25% vs. 2.7%, *p* = 0.005, Fisher’s exact test). Similarly, the presence of high uterine artery PI (defined as >1.5) was more common in the study group than in controls (38.9% vs. 0%, *p* < 0.001, Fisher’s exact test).

The incidence rates of significant proteinuria in the study group and the control group were compared. Significantly more frequent protein loss exceeding 30 mg/dl was observed in the study group compared to the control group (Fisher’s exact test; *p* < 0.001).

### 3.3. Expression of E-Cadherin in the Study and Control Groups

**Figure 1:**
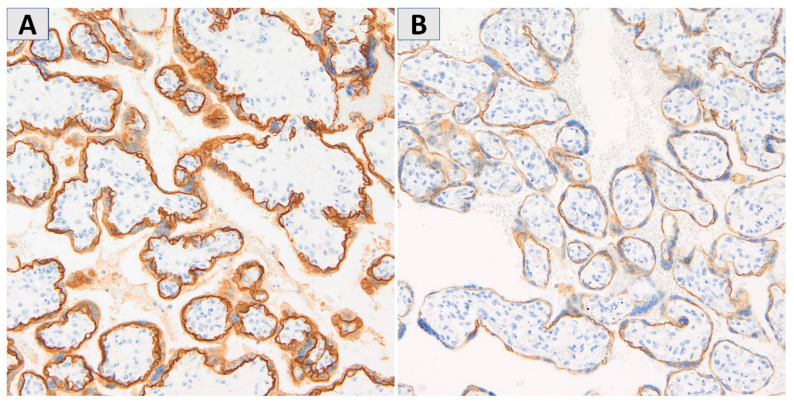
The representative examples of intense, continuous membranous E-cadherin staining (**A**) and very weak, discontinuous E-cadherin staining in the syncytiotrophoblastic basal membrane (**B**). Magnification 200×.

To assess the continuity and intensity of E-cadherin expression, the placenta was divided into maternal, central, and fetal parts. The representative examples of continuous and discontinuous staining patterns in the syncytiotrophoblastic basal membrane are shown in Figure 1.

**Figure 2:**
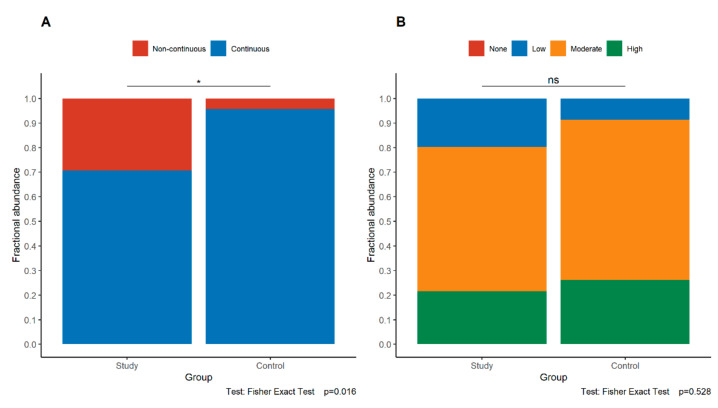
Comparison of the pattern (**A**) and intensity (**B**) of E-cadherin expression in the maternal part of the placenta between the study group and control group. The asterisk depicts statistically significant associations. Abbreviations—ns—not significant.

**Figure 3:**
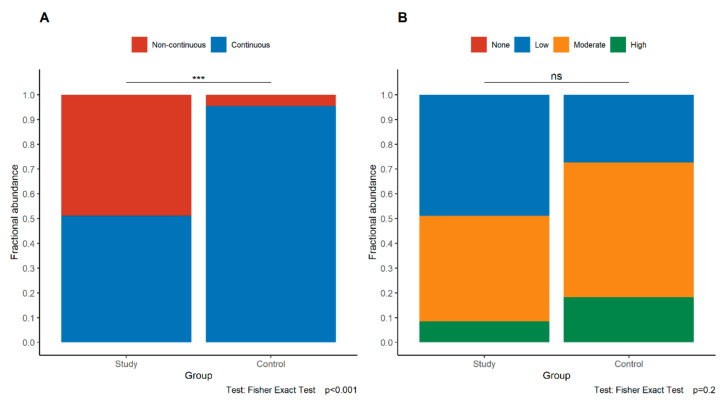
Comparison of the pattern (**A**) and intensity (**B**) of E-cadherin expression in the fetal part of the placenta between the study group and control group. Asterisks depict statistically significant associations. Abbreviations: ns—not significant.

Loss of continuity of E-cadherin expression in the maternal part and the fetal part of the placenta was observed significantly more frequently in the study group than in the control group (maternal part *p* = 0.016, fetal part *p* < 0.001, Fisher’s exact test) (Figure 2A and Figure 3A). These relationships were retained even if only study group cases of gestational age at least 32 weeks were compared with controls (maternal part *p* = 0.037, fetal part *p* = 0.002, Fisher’s exact test).

On the contrary, the intensity of E-cadherin expression in the maternal and fetal parts of the placenta was similar in both groups (maternal part *p* = 0.528, fetal part *p* = 0.2, Fisher’s exact test) (Figure 2B and Figure 3B).

### 3.4. Expression of p120 in the Study and Control Groups

**Figure 4:**
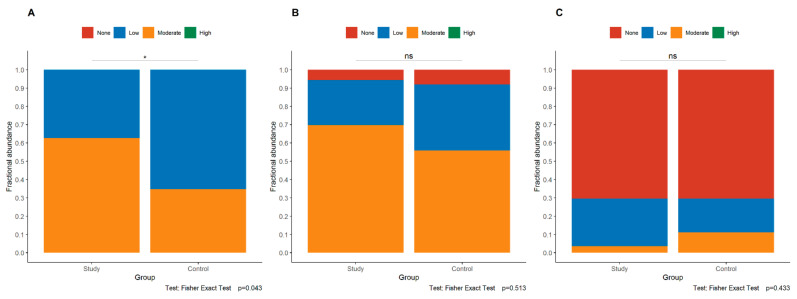
Comparison of cytoplasmatic intensity in trophoblastic cells (**A**) and membranous expression in trophoblast (**B**) and endothelia (**C**) of p120 in the maternal of the placenta. The asterisk depicts statistically significant associations. Abbreviations: ns—not significant.

Expression of the p120 protein was assessed in the maternal, central, and fetal divisions of the placenta. Additionally, in each of these parts, the p120 protein was assessed separately in the cytoplasm, cell membrane of trophoblast cells, and vascular endothelial cells. In the maternal part of the placenta, significantly stronger cytoplasmatic expression of the p120 was observed in the study group compared to in the control group (*p* = 0.043, Fisher’s exact test, Figure 4A), but no statistically significant difference was observed between the study group and the control group in the membranous expression of p120 in the trophoblast (*p* = 0.513, Fisher’s exact test, Figure 4B) and in the endothelium (*p* = 0.433, Fisher’s exact test, Figure 4C). No association was found between the expression of p120 and the abnormalities in uteroplacental circulation on ultrasound.

### 3.5. Loss of E-Cadherin Continuity Is Associated with the Presence of an Early Diastolic Notch in the Uterine Arteries, Maternal Proteinuria, and Lower Apgar Scores

**Figure 5:**
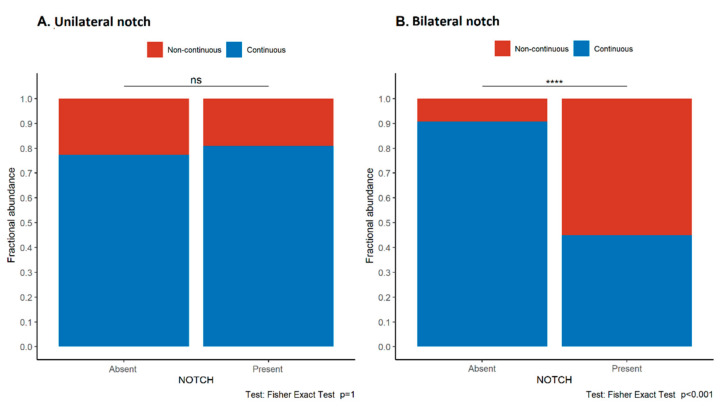
Associations between the pattern of E-cadherin expression in trophoblast and the presence of unilateral (**A**) and bilateral (**B**) early diastolic notch in the uterine arteries in the study group. Asterisks depict statistically significant associations. Abbreviations: ns—not significant.

The continuity and intensity of E-cadherin expression in the syncytiotrophoblast in the maternal, central, and fetal divisions of the placenta were compared with the presence of ultrasound features of uteroplacental malperfusion, the presence of maternal proteinuria, and condition of the newborn (birth weight, birth weight percentile, and Apgar score) for the study group. Loss of continuity of E-cadherin expression in the maternal parts was significantly more frequent in patients with a bilateral but not unilateral early diastolic notch in the uterine arteries as compared to patients without notching (*p* < 0.001, Fisher’s exact test, Figure 5). No such association was found in the study group (Figure S1). No difference was found between the E-cadherin expression pattern in the fetal and central parts of the placenta and the presence of the diastolic notch. A high uterine artery PI did not correlate with E-cadherin expression.

**Figure 6:**
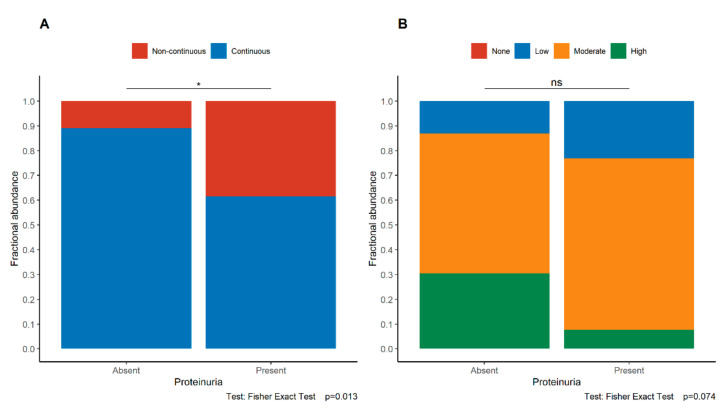
Associations between the pattern (**A**) and intensity (**B**) of E-cadherin expression in trophoblast and the presence of proteinuria in the study group. The asterisk depicts statistically significant associations. Abbreviations: ns—not significant.

In the maternal part of the placenta, a significant correlation between the loss of continuity of E-cadherin expression and the occurrence of proteinuria was found (Fisher’s exact test; *p* = 0.013, Figure 6). In the control group, there was no association between the E-cadherin expression and proteinuria (Figure S2). Moreover, an association between E-cadherin discontinuity and an Apgar score of ≤7 in the newborn was noted (*p* = 0.012). On the other hand, birth weight was not significantly associated with the pattern of E-cadherin expression (*p* = 0.941, Mann–Whitney *U*-test).

## 4. Discussion

In the current study, we demonstrated that the loss of E-cadherin continuity in the syncytiotrophoblastic basal membrane correlates with the presence of an early diastolic notch in the uterine arteries, maternal proteinuria, and lower Apgar scores in newborns. It may indicate that altered E-cadherin expression could be a marker of placental barrier disruption.

Pregnancy-induced hypertension and changes in the spiral arteries result in inadequate nutrition of the fetus [22,23]. The consequences are preterm labor (spontaneous and iatrogenic), cesarean delivery, low birth weight, and a poor general condition of the newborn (low Apgar score) [24,25]. The results of our study confirm that the loss of E-cadherin expression continuity in the syncytiotrophoblastic basal membrane correlates with increased vascular resistance and the presence of early diastolic notches in the flow waveform of the uterine arteries, as well as the presence of proteinuria in patients with PIH/PE and lower Apgar scores in newborns.

Taking into consideration that the syncytiotrophoblast forms the largest interface between the mother and fetus, the discontinuity of E-cadherin expression may indicate the lack of integrity of the fetal–maternal barrier. Moreover, we observed more frequent cytoplasmatic expression of p120 in the study group when compared to the control group. To our knowledge, no studies have described E-cadherin continuity or discontinuity in the basement membrane of syncytiotrophoblast as an immunohistochemical marker of the development of PIH. The altered secretion of active substances from the placenta through the damaged placental barrier into the bloodstream may cause the symptoms present in PIH/PE [26,27]. 

The crucial role of E-cadherin continuity in the basement membrane of syncytiotrophoblast is supported by studies on syncytin 1 [28]. It is a cell–cell fusion protein that promotes the proliferation of cytotrophoblast by regulating the cell cycle and also mediates the fusion of cytotrophoblasts to the syncytium [28]. Under physiological conditions or in the early stages of PE, the low oxygen levels causing hypoxia and oxidative stress have been shown to activate syncytin 1 expression, which accelerates proliferation and cytotrophoblast fusion to replace damaged syncytium [29,30,31]. However, over time, in the later stages of PE, this mechanism is overloaded, and the expression of the syncytin 1 gene decreases [32,33]. This hypoxic environment disturbs syncytial homeostasis through the impaired proliferation and fusion of cells as well as increased cell apoptosis. Studies have shown that in the normal placenta, syncytin 1 expression is mostly detectable in the basal membrane of the syncytiotrophoblast, while in the placentas from patients with PE, expression is limited to the thin apical lining of the microsomal syncytiotrophoblast membrane [7,33]. The improper distribution of syncytin 1 and its inaccessibility at the fusion sites leads to activation of the apoptotic pathway and death of trophoblast cells [7]. The evolution of changes leads to impaired syncytial tightness, which is reflected in the loss of continuity of E-cadherin expression in the basement membrane of the syncytiotrophoblast and causes the release of syncytiotrophoblast extracellular vesicles (STBEV) [34]. It is known that the release of STBEV by syncytium into the mother’s bloodstream is a hallmark of a healthy and normal pregnancy [35]. Until recently, these vesicles were thought to consist only of indifferent debris of trophoblast. Their immunomodulatory activity is highlighted by their role in normal and pathological pregnancies, especially in PE [36]. In PE, STBEVs are secreted in much greater amounts and have pro-inflammatory, anti-aggregation, and procoagulant effects, which leads to endothelial dysfunction and activation of the coagulation system [37]. A significant increase in the release of STBEVs and thus an increase in their concentration in women with PE during labor may also play a role in the exacerbation of maternal symptoms during the perinatal period. STBEV carries a complex of proteins, lipids, and mRNA [38]. In PE, qualitative and quantitative changes were found in the secreted STBEVs. Particularly noteworthy is the reduced content of syncytin 1 [39]. We hypothesize that the abnormal course of EMT leads to ischemia, hypoxia, and a decrease in syncytin 1 gene expression, which results in abnormal function of the maternal–fetal barrier (syncytiotrophoblast) and a loss of E-cadherin continuity. Potentially, measurement of the levels of soluble E-cadherin (sE-cadherin), a peptide degradation product of the E-cadherin, may serve as a serum marker of EMT and reflect the E-cadherin expression status in the placenta. This hypothesis should be tested in future studies [40].

Additionally, in PE, the ischemic placenta secretes excessive anti-angiogenic agents, which leads to endothelial dysfunction, especially in fenestrated endothelial cells, which are found in the brain, liver, and glomeruli. One of the clinical presentations of endothelial injury is proteinuria. In the kidneys, the adhesion between podocytes and endothelial cells of the glomerular capillaries is disturbed, which leads to damage of the filtration barrier [41]. An additional mechanism may include the disruption of the placental barrier reflected by loss of E-cadherin integrity, demonstrated in the current study, which may potentially cause leakage of free fetal hemoglobin into the maternal circulation, resulting in increased oxidative stress and glomerular damage [41].

The presence of an early diastolic notch in the uterine arteries showed a significant correlation with the discontinuity of E-cadherin expression. This event likely leads to damage of the placental barrier, hypoxia due to incorrect placental implantation, and increased vascular resistance reflected by the presence of early diastolic notches in the uterine arteries in the study group. Due to the low number of healthy pregnant women with abnormal flow in the uterine arteries in the control group, it was not possible to perform statistical analysis of the results of the immunohistochemical tests with Doppler examinations in this group. Thus, it remains unclear whether women with uterine flow abnormalities and no PE would have a loss of E-cadherin continuity similar to those seen in women with PE. To make definitive conclusions, it would be necessary to gather a larger study group of these specific types of patients.

Unequivocal changes due to PIH are not found on histopathological examination. Changes in the villi of the placenta, their premature maturation, and infarcts or vascular changes in the myometrium also occur in other pathologies: premature delivery, stillbirth, fetal growth restriction, or premature separation of the placenta [42,43,44]. In a study by Sebire et al., they report a threefold increase in the recognition of PIH changes when the pathologist knew the diagnosis compared to when they did not know the diagnosis, which proves the lack of objectivity in their assessments [45]. These findings suggest that there are no sensitive or specific histopathological changes for PIH. The assessment of E-cadherin continuity could be a helpful marker in the study of the placenta in PIH/PE.

Our study has several limitations. The main limitation is the small sample size and the fact that no power analysis was performed before the study (all eligible patients who agreed to participate were enrolled). Moreover, some differences, especially in the duration of pregnancy, exist between the study and control cohorts, which might have led to bias in the placental analysis. No soluble markers of pre-eclampsia (e.g., s-Flt1/PIGF ratio) were analyzed. Future studies on this topic should address the association between the concentration of soluble agents contributing to the development of PIH/PE and the pattern of E-cadherin expression in the placenta. Moreover, it is unknown if a placental disruption in other gestational diseases is associated with similar E-cadherin alterations; thus, our results cannot be generalized to the entire pregnant population.

To conclude, our study suggests that the discontinuity of placental E-cadherin expression is the potential marker of placental barrier damage and may contribute to its increased permeability in patients with PIH/PE. 

## Figures and Tables

**Table 1:** Comparison of basic parameters between groups. Abbreviations: D—daughter; S—son; L—live; T—term; P—preterm; cc—cesarean section; n/a—not available.

		Study Group (*n* = 55)	Control Group (*n* = 37)	*p* Value
Gravida	1	*n* = 37	*n* = 20	
	2	*n* = 11	*n* = 12	0.367
	≥3	*n* = 7	*n* = 5	
	n/a	*n* = 0	*n* = 0	
Newborn	D/L/T	*n* = 16	*n* = 20	
	D/L/P	*n* = 18	*n* = 0	
	S/L/T	*n* = 9	*n* = 16	<0.001
	S/L/P	*n* = 10	*n* = 0	
	n/a	*n* = 2	*n* = 1	
Delivery	cc	*n* = 44	*n* = 7	
	vaginal	*n* = 10	*n* = 29	<0.001
	n/a	*n* = 1	*n* = 1	
Apgar score	>7	*n* = 38	*n* = 30	
	≤7	*n* = 10	*n* = 0	<0.001
	n/a	*n* = 7	*n* = 8	
Gestational age	<32 weeks	*n* = 8	*n* = 0	
	≥32 weeks	*n* = 47	*n* = 37	0.015

## Data Availability

Data are available from the authors upon reasonable request.

## Supplementary Materials

The following are available online at https://www.mdpi.com/article/10.3390/jcm11030668/s1, Figure S1—Associations between the pattern of E-cadherin expression in trophoblast and the presence of unilateral (A) and bilateral (B) early diastolic notch in the uterine arteries in the control group. Figure S2—Associations between the pattern (A) and intensity (B) of E-cadherin expression in trophoblast and the presence of proteinuria in the control group.
