# Supplementary material for: Loss of E-Cadherin Staining Continuity in the Trophoblastic Basal Membrane Correlates with Increased Resistance in Uterine Arteries and Proteinuria in Patients with Pregnancy-Induced Hypertension
Source: J Clin Med. 2022 Jan 27;11(3):668. doi: 10.3390/jcm11030668 (PMC8836559; doi:10.3390/jcm11030668)
Supplement: Supplementary file 1 [file jcm-11-00668-s001.zip › supplementary_figures.pdf]

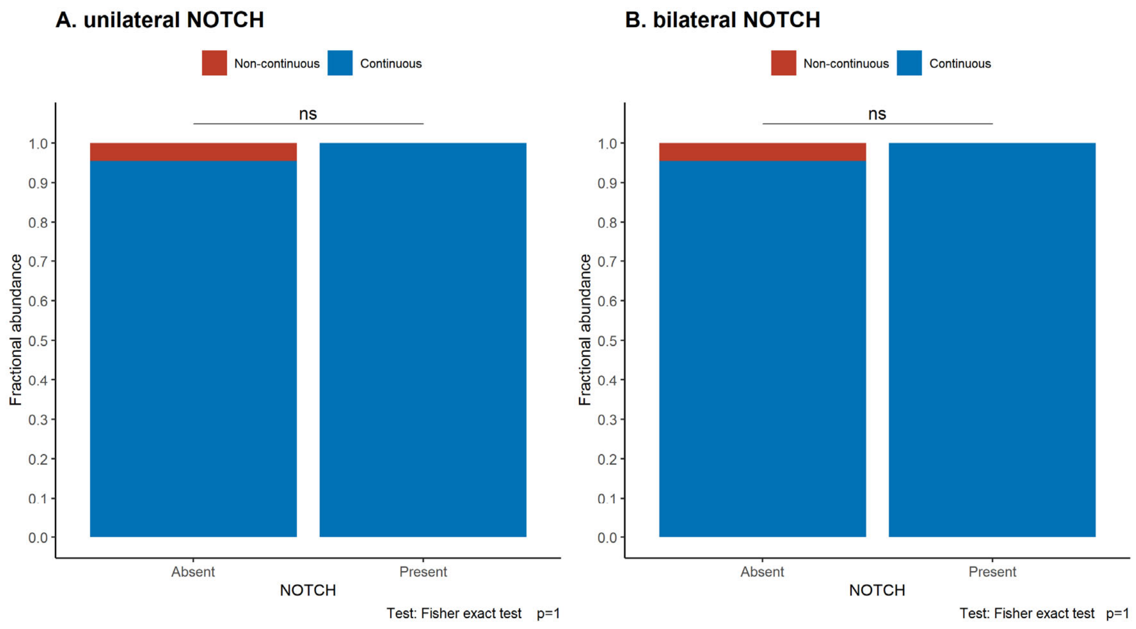

Figure S1. Associations between the pattern of E-cadherin expression in trophoblast and the presence of unilateral (**A**) and bilateral (**B**) early diastolic notch in the uterine arteries in the control group. Abbreviations – ns – not significant.

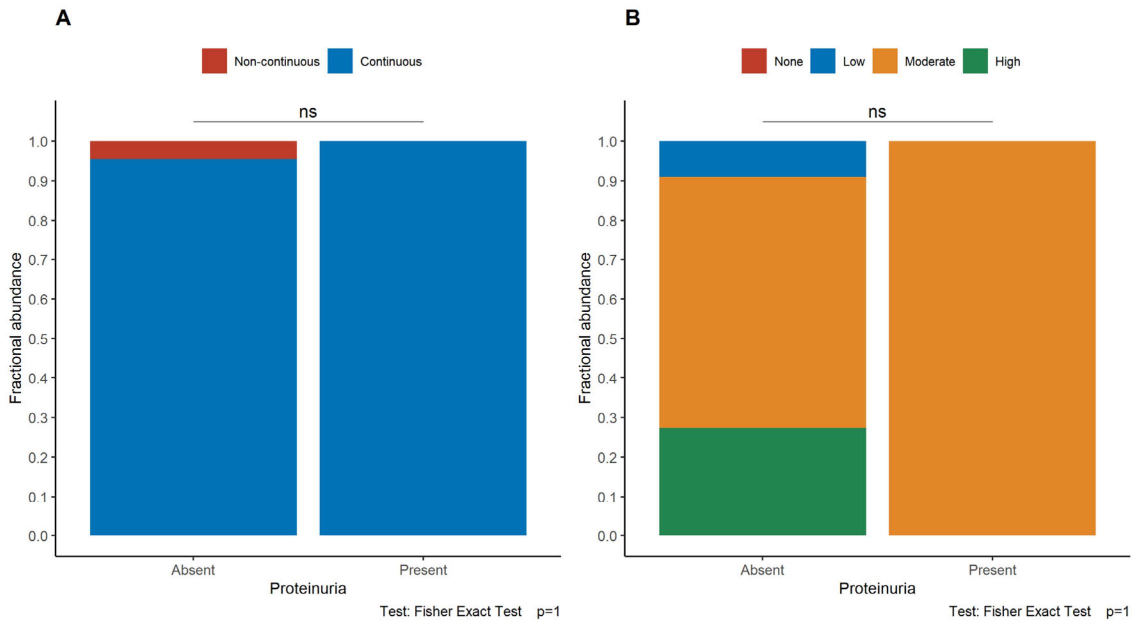

Figure S2. Associations between the pattern (**A**) and intensity (**B**) of E-cadherin expression in trophoblast and the presence of proteinuria in the control group. Asterisks depict statistically significant associations. Abbreviations – ns – not significant.
